# Effect of Birch Sawdust Hydrolysis on Chemical Characteristics, Aggregation, and Surface Activity of Extracted Soda Lignin

**DOI:** 10.3390/polym17111455

**Published:** 2025-05-23

**Authors:** Galia Shulga, Brigita Neiberte, Valerija Kudrjavceva, Anrijs Verovkins, Arturs Viksna, Sanita Vitolina, Julija Brovkina, Talrits Betkers

**Affiliations:** 1 Latvian State Institute of Wood Chemistry, Dzerbenes 27, LV-1006 Riga, Latvia; brigita.neiberte@kki.lv (B.N.); valerija.kudrjavceva@kki.lv (V.K.); anrijs.verovkins@kki.lv (A.V.); sanita.vitolina@kki.lv (S.V.); yuli@inbox.lv (J.B.); betalrits@gmail.com (T.B.); 2 Faculty of Medicine and Life Sciences, Latvian University, Jelgavas 1, LV-1004 Riga, Latvia; arturs.viksna@lu.lv

**Keywords:** wood sawdust, alkaline hydrolysis, pulping, soda lignin, aggregation, surface tension, structural complementarity

## Abstract

Various pretreatment methods, often employed in wood biorefineries, aim to disrupt the wood architecture, thereby enhancing the efficiency of hemicellulose extraction for increasing the production of bio-ethanol, bio-gas, and bio-oil, as well as improving the pulping process. Pretreatment for the pulping process has advantages such as enhanced yield in biorefined products and reducing chemicals and energy consumption. This study examined the effect of an alkaline hydrolysis of birch sawdust on the chemical composition, aggregation ability, and surface activity of soda lignin obtained by soda pulping. The alkaline hydrolysis of birch sawdust led to a remarkable removal of hemicellulose and reduced its mechanical strength. The resorption of lignin fragments on the lignocellulosic matrix during the hydrolysis was observed. The soda pulping of the original and the treated sawdust was carried out under laboratory conditions at 165 °C for 90 min, using 4.5% sodium hydroxide. A higher yield of soda lignin and pulp was obtained from the treated sawdust. The reduced content of acidic and methoxyl groups in the chemical composition of the soda lignin from the hydrolyzed sawdust was explained by the predominance of polycondensation reactions in forming its primary structure. The changes in size and zeta potential values of the formed lignin particles, as well as in the modality of the size distribution with decreasing pH, were studied. The early-proposed suggestion about the existence of structural complementarity in the formation of the ordered lignin supermolecular structures has been testified. The higher surface activity at the air–water interface for the soda lignin extracted from the hydrolyzed sawdust, compared to the lignin from the original residue, was mainly attributed to a lower content of the acidic groups in its chemical composition, shifting the hydrophilic–hydrophobic balance of its structure toward hydrophobicity.

## 1. Introduction

It is known that wood biomass consisting of cellulose, hemicelluloses, and lignin is a widely available and renewable feedstock for obtaining paper, biofuels, and bioethanol, along with the development of new value-added products [1,2,3]. Lignin, an integral part of wood biomass, is one of the most abundant aromatic sources. Otherwise, it is the third most abundant biopolymer resource available for humans (after cellulose and chitin).

Lignin is a superbranched, phenylpropane-based amorphous polymer, in which its phenylpropane chains, bonding with ester, ether, and carbon–carbon linkages, form the fractal structures [4]. The formation of lignin in wood proceeds due to free radical polymerization processes of monolignols leading to the formation of the lignin’s structural units such as p-hydroxyphenyl, guaiacyl, and syringyl [5]. The content of lignin’s structural units varies by wood species; for example, hardwood is characterized by a high content of syringyl propane units, while softwood contains more guaiacyl propane units. Hardwood and softwood also differ in lignin content: hardwood contains approximately 20–25% lignin, but in softwood, the lignin content is higher and can reach up to 30%. Typical functional groups of lignin are aliphatic and aromatic hydroxyl, methoxyl, carboxyl, and carbonyl groups. The space structure, different functional groups, and various linkages between the structural units contribute to lignin’s complexity and heterogeneity, creating difficulties for its quantitative and qualitative extraction from a lignocellulosic matrix of wood and plants and complicating the establishment of the structure and morphology of lignin in situ [6,7,8,9,10,11,12,13].

The extraction efficiency of lignin and its characteristics, such as chemical composition, structure, and molecular weight, depend on several factors, including biomass origin, extraction procedure and its peculiarities, drying features, etc. Oxidation, condensation, polymerization, and other chemical reactions can affect the lignin extraction yield from the lignocellulosic matrix [8]. Extraction methods and conditions can be decisive factors for the potential valorization of the obtained lignin [14]. The valorization of lignin presents an excellent opportunity due to its abundance, low cost, and beneficial properties, such as biodegradability, thermal stability, and high carbon content [15]. As the most abundant renewable aromatic biopolymer, lignin can effectively serve as an alternative to petroleum-based aromatic polymers, fostering a more sustainable future.

Technical lignin extraction from wood occurs during different pulping processes used for cellulose production. It is known that approximately 50–70 million tons of technical lignins as a by-product are produced at pulp and paper mills per year. For the most part, the lignins are used for power generation at the mills, and the formed wastewater is a main factor that negatively affects the environment. Only 1–2% of the produced lignin is utilized for making value-added products [16]. Given lignin’s significant economic and industrial potential, extracting high-purity lignin from lignocellulosic biomass is essential for its effective subsequent usage. Technical lignins and lignin-based products are used as binders, plasticizing and dispersing agents, phenol substitutes in phenol–formaldehyde resins, bio-additives to plastics and composites, carbon fiber precursors, etc. [15,17,18,19,20]. They usually have a purity of at least 70%, which corresponds to a Klason lignin content. Klason’s lignin value in commercial products is more than 85%. The purity of the extracted lignin has a decisive impact on its potential valorization for obtaining new value-added bioproducts [21,22,23,24,25].

The technical lignins obtained by alkaline pulping can be classified depending on the presence of sulfur, namely, the sulfur-containing lignin—Kraft and sulfur-free lignin—soda [26]. Soda lignin is a by-product of the cooking of hardwood and herbaceous plants [27,28]. The industrial pulping proceeds by heating the lignocellulosic biomass in a pressurized reactor at 140–170 °C in the presence of 10–16 wt% alkali (ordinary sodium hydroxide) [29]. The soda pulping of the biomass offers low-cost methods for chemical recovery and more efficient technologies for treating effluent. Its use may also be linked to less stringent environmental regulations regarding effluent discharge in certain countries. Additionally, the extracted soda lignin has a chemical composition and structure that closely resembles that of native lignin [29,30]. Compared with Kraft pulping, the major advantage of soda pulping is the absence of sulfur, which is a reason for the unpleasant odor during the production of cellulose. Soda lignin is produced on a commercial scale by the world’s major producer, Green Value SA, with an annual capacity of about 10,000 tons of high-purity lignin known as Protobind [30]. The molecular weight of the soda lignin is lower than that of kraft lignin and lignosulfonates [31,32,33]. Due to its chemical composition and structural features, soda lignin is used as an adsorbent [34], for obtaining a compatibilizer for wood–plastic composites [35], polyelectrolyte membrane for UV-absorbance [36], antibacterial lignin nanoparticles for packaging [37], an asphalt modifier [38], etc.

The alkali lignins in aqueous and organic solutions have an ordered structural organization that affects many technological processes, including wood delignification [39]. Due to the presence of hydrophobic aromatic rings and propanoid chains containing polar and hydrophilic functional groups, lignin molecules have an amphiphilic character and are capable of self-organizing in solutions. This is evident in the formation of supermolecular structures resulting from the self-aggregation of lignin molecules and the self-assemblies of lignin aggregates [40]. The study of aggregation processes of soluble lignins in solutions to obtain non-covalent assemblies with defined structures is important, as it can present new opportunities for scientific advancement in creating innovative, value-added products with specific applications, improving technologies, and contributing to the advancement of the bioeconomy. Knowledge of the self-organization behavior of lignin can provide significant structural information to optimize pulping or extraction processes, to choose lignin modification reactions, to increase the efficiency of biorefining, and to create new value-added products [41].

There are many research works devoted to the study of the aggregation of various lignins in different solutions and mechanisms of their supermolecular structure formation [39,40,41,42,43,44,45,46,47,48,49,50,51]. The generalization of the obtained points of view on the structuring processes in lignin solutions can be described in such a manner. Soluble lignins have some levels of structural organization in solution, namely, self-organization of lignin molecules affecting their conformational state (shape), self-aggregation of the organized lignin molecules in the form of small particles as small building blocks, and self-assembly of these lignin blocks leading to the formation of large supermolecular structures such as colloidal particles [42]. Until now, there has been no common opinion on the shape and size of lignin molecules in solution. It has been proposed that the lignin molecule can have various shapes, such as spherical, ellipsoidal, disk-shaped, etc., wherein lignin molecule sizes vary from 3 to 10 nm [44]. The spherical arrangement of the lignin molecules in solution with the appearance of ellipsoidality is considered more rational due to its smallest specific surface area in contact with water, which responds to the lowest free energy. The self-organized lignin molecules in an aqueous solution may be imagined as having a “core–shell” structure [42,51], a “core” of which is formed by aromatic rings via non-covalently bonded π-π stacking interactions, strengthened by hydrogen bonding and hydrophobic forces, while the hydrophilic part of the lignin molecule forms a “shell” structure. 

The possibility of the organized lignin molecules to form non-covalent assemblies depends on many factors, including their chemical composition and molecular weight as well as on solution properties such as pH, concentration, solvent quality (good, theta, poor), temperature, stirring or mixing velocity, etc. [41,50]. The building up of the supermolecular lignin structures proceeds due to electrostatic, π-π stacking interactions, H-bonding, and hydrophobic interactions between the functional groups disposed of in the “shell” part of the self-organized lignin molecules [49]. According to the proposed mechanism, the formation of the lignin supramolecular structures occurs through the layer-by-layer deposition of the lignin molecules on the surface of the highest molecular weight lignin molecule located in the center of this formation. Because the core is formed first, it can dictate the subsequent growth of the self-aggregates, which occurs from the center outward, like the formation of “onion-like” structures. It was assumed that self-assembly is a spontaneous process having a cooperative character [40,42,50].

With increasing concentration of lignin in solution, the shape of its supermolecular structure changes from spherical or ellipsoid to cylindrical, similar to the conversion of spherical micelles to cylindrical or rod-like ones with increasing surfactant concentration. It was stated that the lignin self-assembly formation has a selective character proceeding via deconstruction and reconfiguration of the previously formed supermolecular structures [40,48].

Due to their amphiphilic nature, soluble lignins are surface active at various interfaces. Surface active properties of lignin are very important from an application point of view, because they are widely exploited in practice. According to the analysis of the patenting activity in recent years, the utilization of various lignins for obtaining surface active agents (stabilizers, emulsifiers, detergents, and dispersants for the treatment of asphalt, latex, soap, cement, clay, etc.) takes first place in comparison with their usage in resins, composites, and concrete, as well as for obtaining low-molecular chemicals [52,53]. Soluble lignin molecules can easily adsorb at various interfaces, creating mono- or multi-layers with viscoelastic or condensed structure and thereby reducing interfacial tension; herewith, the charged fragments of lignin molecules are in an aqueous phase, while the hydrophobic ones are disposed into a non-aqueous phase. It is reported that lignin adsorbs at the interfaces as aggregates [54]. The adsorption process of lignin molecules at the interface depends on lignin chemical composition, molecular weight, concentration, ionic strength, and pH [55].

One of the main characteristics of traditional surfactants is the critical micelle concentration (CMC). Considering the polymer nature of the lignin molecules, a definition such as critical aggregation concentration (CAC) is more suitable. The CAC values for soluble lignins vary widely, ranging from 0.05 g/L to 0.38 g/L, and are often dependent on the experimental conditions and the technique used for CAC measurement [56].

Biomass pretreatment is important in wood and other lignocellulosic materials’ biorefinery. Various pretreatments of the biomass, such as steam explosion, autohydrolysis, acid hydrolysis, ammonia treatment, chemical pulping, solvent extraction, etc., increase the reactivity of the biomass components, making the extraction process easier and more efficient [32,57,58]. For an increase in the intensity and selectivity of the delignification process during wood pulping, and a decrease in the consumption of pulping reagents, water, and heat consumption, the pretreatment of wood biomass with alkaline solutions (NaOH, Ca(OH)_2_, NH_4_OH) along or together with other additives is used [25,27,31]. The main aim of the initial stage of the alkaline pretreatment is the partial destruction of the lignocellulosic matrix to facilitate the removal of hemicelluloses and lignin during pulping.

Previous works, in which the influence of the alkaline pretreatment of lignocellulosic materials on their soda pulping was examined, focused mainly on the impact of the pretreatment on the removal of hemicellulose, the yield of pulp, its quality, and the characteristics [11,59,60,61]. However, there is insufficient information on how alkaline pretreatment affects the characteristics and properties of the soda lignin extracted from black liquor, especially in its ability to aggregate in aqueous media [26,62,63].

This research aimed to compare the chemical characteristics, aggregation ability, and surface activity of soda lignin obtained by soda pulping of original and alkali-hydrolyzed wood residue.

## 2. Materials and Methods

### 2.1. Materials

#### 2.1.1. Hydrolysis

Birch sawdust, representative of a residue of the mechanical processing of birch wood, was supplied by the company Latvijas Finieris Ltd. (Riga, Latvia). The fractionated sawdust with particles ≤ 250 μm was characterized by elemental analysis (Elementar Analysensysteme, Langenselbold, Germany) and wood composition according to Klason and Kürschner chemical procedures for lignin as per the TAPPI T222 om-11 standard [64] and the TAPPI T203 cm-99 standard [65], respectively. The hemicellulose content was determined according to the TAPPI T264 om-97 standard [66]. The elemental composition of the birch wood sawdust was the following: 48.1% C, 5.9% H, 45.7%, 0.2% N, and 0.1% S. The water-soluble extractives (2.2%) and ash (0.4%) content were determined according to Zakis [67].

The birch sawdust treatment was carried out under laboratory conditions: 0.05% to 0.50% NaOH water solutions, temperature—90 °C, duration—5 h, with hydromodulus (a sawdust-to-water weight ratio)—1:20, using three-neck glass flask, equipped with a return condenser, a thermometer, a stirrer, and a water bath. After the treatment, the hydrolyzed sawdust was isolated from the hydrolysate by filtration, washing, and drying at 60 °C for 48 h and then at 105 °C for a short time in a heating cabinet.

The yield of the released extractive products during the alkali pretreatment was calculated according to Equation (1).

Extractive products, % = (m_o_ − m_h_)/m_o_ × 100, (1)

where m_o_—weight of oven-dried original sawdust, g

m_h_—weight of oven-dried hydrolyzed sawdust, g

The low-molecular reagents were purchased from Sigma-Aldrich (Steinheim, Germany) and were used as received without further purification.

#### 2.1.2. Pulping

Soda lignin samples were obtained by pulping both origin and alkali-treated birch sawdust in a laboratory reactor under the following conditions: NaOH concentration—4.5%, hydromodulus—1/7, duration—90 min, temperature—165 °C, and pressure—0.6 MPa. The purification of the precipitated lignin samples was achieved through three repeated dissolutions in a 0.1 M NaOH solution, followed by precipitation with concentrated sulfuric acid. Each time, the precipitate obtained on the filter was carefully washed with diluted sulphuric acid (1%) and hot water. After triple dissolution/precipitation, the Klason lignin content in the lignin samples was determined.

The yield of the soda lignin obtained during the sawdust pulping was calculated according to Equation (2).

Soda lignin, % = m_sl_/m_o_ × 100, (2)

where m_o_—weight of oven-dried sawdust, g

m_sl_—weight of oven-dried extracted lignin, g

The pulp yield obtained was calculated according to Equation (3).

Pulp, % = m_p_/m_o_ × 100, (3)

where m_o_—weight of oven-dried sawdust, g

m_p_—weight of oven-dried pulp, g.

### 2.2. Methods

#### 2.2.1. Elemental and Functional Analysis

The content of Klason lignin was determined according to TAPPI T222 om-02 standard [64]. The chemical composition of lignin samples was determined by elemental analysis (Elementar Analysensysteme GmbH, Langenselbold, Germany). Functional group analysis was carried out using the Fibok–Shvappakh method for methoxyl groups, the acetylation method for aliphatic hydroxyl groups, the interaction with hydroxylamine hydrochloride for carbonyl groups, and potentiometric and conductometric titration for phenolic hydroxyl and carboxyl groups, according to Zakis [67]. The elemental and functional analysis for each lignin sample was performed three times.

#### 2.2.2. Fourier Transform Infrared Spectroscopy 

The FT-IR study was performed using a Perkin Elmer Spectrum One apparatus (Perkin Elmer, Waltham, MA, USA) at the range of wavenumbers from 4000 to 450 cm^−1^ (30 scans), at a resolution of 4 cm^−1^. The tablets were prepared by mixing 20 mg of a sample with 200 mg of KBr, and then the tablet compression method was used.

#### 2.2.3. UV/Visible Spectroscopy

The study of the hydrolysate formed after the pretreatment of birch sawdust was performed at wavelengths of 280 nm and 490 nm, using a Genesys 10UVUV/VIS spectrophotometer (Termo Fisher Scientific, San Jose, CA, USA). These wavelengths correspond to the presence of different wood-originated substances in the hydrolysates; namely, absorbance at 490 nm was related to a mixture of low molecular wood destructive products such as fragments of hemicelluloses, lignin, lignin–carbohydrate complexes, and extractives, but absorbance at 280 nm was related only to lignin and lignin-like fragments [68].

#### 2.2.4. Potentiometric Titration

The titration curves were obtained using a Radiometer Analytical Titrolab 90 with autoburette ABV 901 (Radiometer Analytical SAS, Villeurbanne Cedex Lyon, France) at 25 °C. For the adjustment of pH values, 0.1 M NaOH and 0.1 M HCl solutions were used. For the preparation of lignin aqueous solutions, double-distilled water was used.

#### 2.2.5. Size and Zeta Potential

The size and zeta (ζ) potential of lignin particles were determined in aqueous solutions using a ZETASIZER NANO ZS Malvern Instrument (Malvern, UK) at 25 °C using a 0.01% alkaline soda lignin solution. This aqueous solution was obtained by diluting a 0.1% soda lignin solution prepared in 0.01 M NaOH. The pH of lignin solutions was changed by adding 0.1 M HCl. After achieving the defined pH value, before measuring, the solutions were left for 15 min at room temperature. The obtained size distribution graphs represented the dependencies of the relative intensity of scattered light on the hydrodynamic diameter of the lignin particles at different pH values of aqueous solution. The intensity area (%) showed the contribution of a particle size mode to the intensity of the scattered light. Tree replicates were made for each sample, and size and zeta potential values were presented as average arithmetic values.

#### 2.2.6. Surface Tension

The surface tension at the air–water interface (σ) was measured by the Wilhelmy plate method using the digital Tensiometer K9 (KRUSS GmbH & Co. KG, Hamburg, Germany) at 25 °C. Surface tension values were determined for each pH value of a lignin aqueous solution 24 h after its storage at room temperature. The pH values were regulated by adding 0.1 M HCl. The aqueous solutions with various concentrations were obtained by diluting a 0.1% soda lignin solution to avoid errors during aqueous solution preparation. Experiments were prepared three times for each sample and presented as average arithmetic values.

#### 2.2.7. Milling

The milling of birch sawdust was carried out with a planetary ball mill (Retsch, Haan, Germany) and sieved using a mill “Pulverisette 0” (Frisch GmbH, Idar-Oberstein, Germany). Time of milling—10 min, rotation speed—300 rpm.

## 3. Results

### 3.1. Hydrolysis of Birch Sawdust

**Figure 1:**
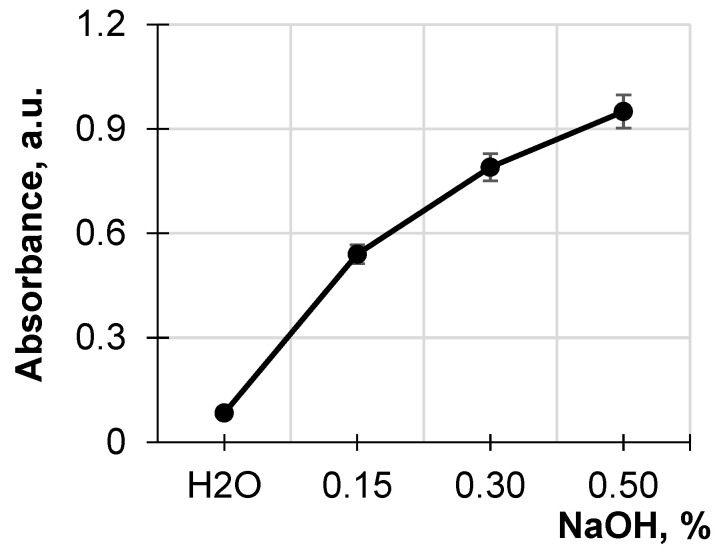
Dependence of absorbance at 490 nm of the hydrolysates on NaOH concentration.

Figure 1 shows the absorption of the obtained hydrolysates of the original sawdust at 490 nm, depending on the NaOH concentration, compared to the absorption of the hydrolysate obtained by treating the sawdust with water. It has been seen that the destruction of the lignocellulose matrix increases with increasing alkali concentration. A comparison of absorption at 490 nm for the hydrolysates for the original sawdust treated with a NaOH solution of various concentrations and water shows a five- to ten-fold increase in absorption for the alkaline hydrolysates compared to that from the aqueous solution. The remarkable increase in the absorption associated with the presence in the hydrolysates of low-molecular water-soluble wood destructive products such as hemicelluloses, lignin, lignin–hemicellulose complexes, and wood extracts indicates the pronounced destruction of the lignocellulose matrix during the alkali treatment. This treatment of the sawdust was accompanied by acidification of the alkaline hydrolysates, which was a sequence of the simultaneous release of low-molecular acids such as acetic, formic, and uronic, which are the products of lignocellulosic matrix destruction [5].

**Figure 2:**
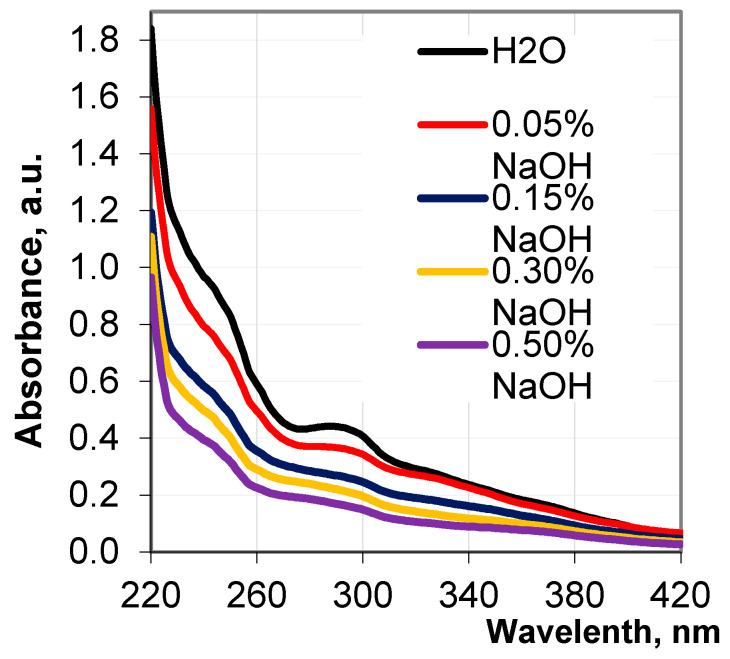
UV spectra of the hydrolysates depending on NaOH concentration.

In Figure 2, the UV curves of the obtained hydrolysates represent the integrity of the absorption bands in the form of strongly pronounced maxima and weakly pronounced shoulders, which testify to the presence of different phenylpropane units in the released lignin fragments. The pronounced shoulders at 230, 245, 260, and 320 nm, and maximum at 287 nm, on the absorbance curves specify the presence of biphenyl derivatives, as well as aromatic structures, containing non-etherified phenolic hydroxyl groups, the hydroxyl groups conjugated with carbonyl groups, and aromatic carboxyl groups in the revealed lignin fragments. It has been seen that with an increase in the alkali concentration from 0.05% to 0.5%, the UV adsorption of the obtained hydrolysates and the height of the peaks at 287 nm remarkably decrease and become flatter. Such a change in the shape of the peak indicates an increase in the concentration of low molecular lignin fragments in the hydrolysates. This is accompanied by a reduction in the values of the extinction coefficient of the hydrolysates from 2.73 g^−1^ cm^−1^ to 1.83 g^−1^ cm^−1^.

The pronounced decrease in the absorbance of the hydrolysates, associated with the progressive destruction of the lignocellulosic matrix with an increase in the alkali concentration, can be explained by the resorption of the released lignin species on the lignocellulosic matrix due to physicochemical interactions in the alkaline medium. This phenomenon has been observed early during wood pulping [39,69,70,71].

**Figure 3:**
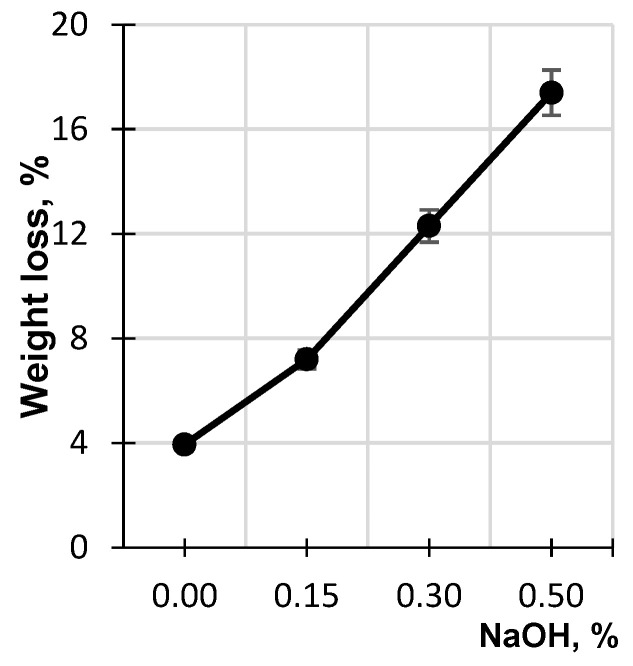
Effect of NaOH concentration on weight loss of birch sawdust.

Figure 3 shows the dependence of birch sawdust weight loss, which was found as the difference between the initial weight of the sawdust and its weight after the hydrolysis, on the concentration of the alkaline solution. It is evident that the higher the concentration of the alkaline solution, the higher the weight loss of the lignocellulosic matrix. At an alkali concentration of 0.5%, the sawdust weight loss reaches 17.4%, which indicates its remarkable hydrolytic destruction.

**Figure 4:**
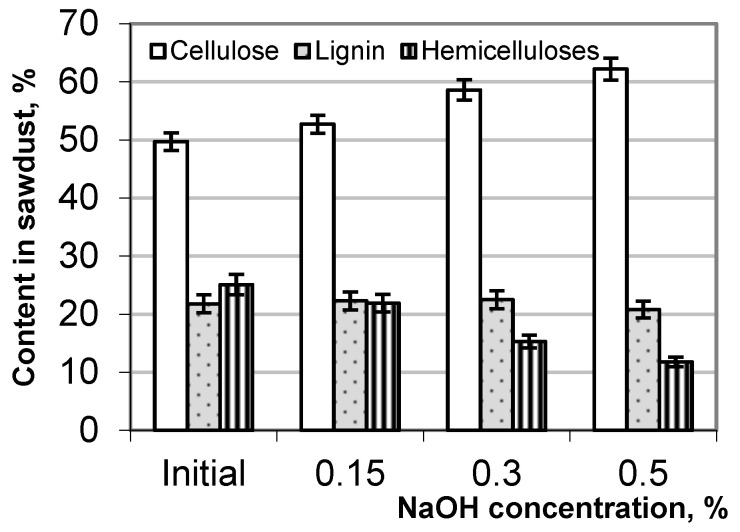
Content of cellulose, hemicellulose, and lignin in birch sawdust before and after the alkaline treatment.

The evaluation of the hydrolytic destruction of the birch wood residue due to the alkali treatment involved analyzing changes in its chemical composition, particularly regarding cellulose, lignin, and hemicellulose. The content of the main wood components before and after the alkaline treatment of the sawdust is presented in Figure 4. With an increasing NaOH concentration, the relative content of cellulose in the hydrolyzed sawdust increases up to 25%, but the content of hemicellulose decreases almost twice relative to its content in the original sawdust. The change in the relative content of lignin in the alkali-treated sawdust is expressed to a lesser extent than cellulose and hemicellulose, which may be associated with the resorption of lignin on the cellulosic surface.

**Figure 5:**
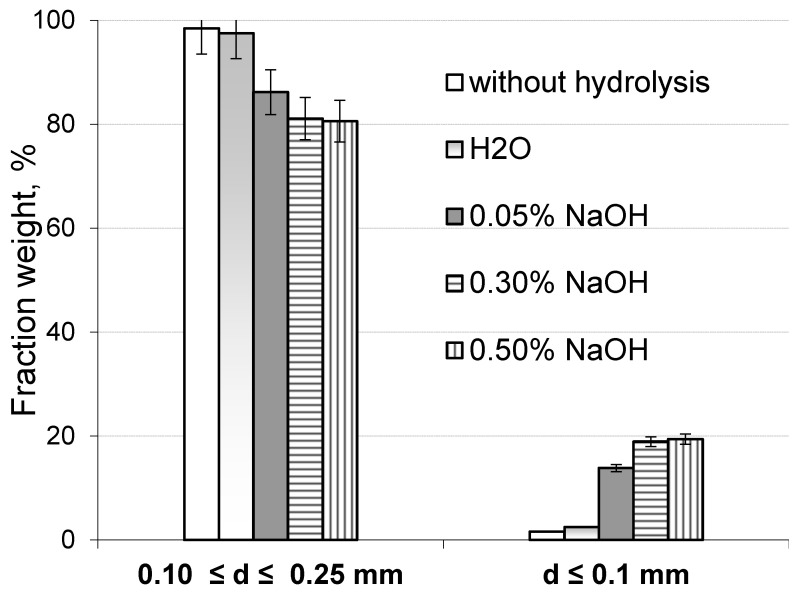
Content of fractions of original and hydrolyzed sawdust depending on NaOH concentration.

Furthermore, the extent of the hydrolytic degradation of the lignocellulosic matrix was assessed by analyzing the fractional composition of both the original sawdust and the sawdust treated with a 0.5% NaOH solution after milling in a planetary mill. The component composition changes in the lignocellulosic matrix of the treated sawdust led to a reduction in its mechanical strength. According to Figure 5, as the alkali concentration increases from 0.15% to 0.50%, the weight of fine particles smaller than 0.1 mm in the milled hydrolyzed sawdust fraction—comprising particles less than 0.25 mm and larger than 0.1 mm—increases from 8 to 12 times compared to the same particle content in the original milled sawdust. It is assumed that the significant increase in the finest particle content in the hydrolyzed sawdust is attributed to the essential removal of hemicellulose from the lignocellulosic matrix as the concentration of sodium hydroxide in aqueous solution rises.

Therefore, based on the generalization of the results obtained, it can be concluded that the highest extent of hydrolytic destruction of the lignocellulose matrix occurs when birch sawdust is treated with a 0.5% NaOH solution.

### 3.2. Chemical Characteristics of Lignin Samples

**Figure 6:**
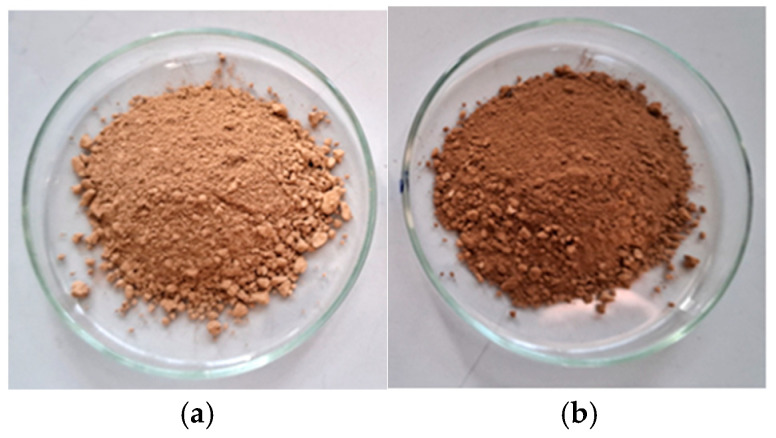
Extracted soda lignin samples from the original (**a**) and the pretreated sawdust (**b**).

The soda lignin samples from the origin sawdust (SLO) and the sawdust hydrolyzed with 0.5% NaOH solution (SLH) (Figure 6) were obtained by the soda pulping of the birch wood residue. Table 1 shows the chemical composition of the obtained soda lignin samples and the yield of the obtained biorefined products—soda lignin and pulp. The results indicate that the yield of the soda lignin and the pulp is higher for the hydrolyzed sawdust than for the original one. This difference amounts to 2.8% and 2.9% for pulp and lignin, respectively.

According to Table 1, the soda delignification of the hydrolyzed sawdust, compared to the delignification of the original sawdust, leads to a decrease in the content of both phenolic hydroxyl and carboxyl groups in the lignin sample. A decrease in the content of the acidic groups in the soda lignin from the hydrolyzed sawdust is accompanied by its more pronounced demethoxylation, namely, the content of methoxyl groups in the soda lignin obtained from the hydrolyzed sawdust (SLH) is 15.1% less than in the lignin from the original sawdust (SLO).

The total yield of the obtained biorefined products derived from the treated sawdust, namely, pulp and lignin, is 5.7% higher than in the case of delignification of the original birch residue; herewith, the pollution going into the environment with the water-soluble destructive products resulting from the pulping of the treated sawdust is lower. A decrease in the content of acidic, namely, phenolic hydroxyl and carboxyl groups along with methoxyl groups in the SLH chemical composition may indicate the prevalence of polycondensation reactions that proceeded between the released lignin molecules over their cleavage reactions during the delignification of the hydrolyzed sawdust.

**Figure 7:**
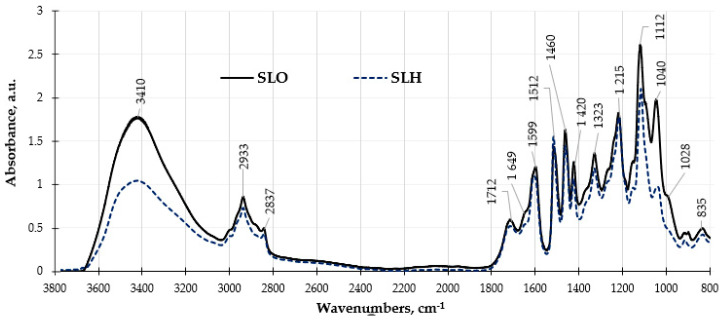
FTIR spectra of soda lignin samples obtained from original and pretreated sawdust.

The changes in the chemical compositions of the lignin samples were consistent with the results of the obtained FTIR spectra analysis presented in Figure 7. In both FTIR spectra, the bands at 1323 cm^−1^ and 835 cm^−1^ are assigned to syringyl rings and are characteristic bands for hardwood lignin.

The significantly lower absorbance band observed at 3410 cm^−1^, which is associated with hydrogen-bonded hydroxyl groups found in phenolic and phenyl propane aliphatic structures, along with the bands at 2933 cm^−1^ and 2837 cm^−1^ due to C–H stretching vibrations in methoxyl groups in the FTIR spectrum of SLH, indicates a reduced presence of these functional groups in this sample compared to SLO. This observation further confirms that the structure of SLH is more condensed than in the case of SLO. The FTIR peaks in the range of 1712–1595 cm^−1^ are assigned to the presence of unconjugated and conjugated carbonyl and carboxyl groups in lignin. Within this range, SLO has a higher absorbance than SLH, which may indicate a lower content of these functional groups in the lignin sample obtained from the hydrolyzed sawdust, which is consistent with the results presented in Table 1. In this range, SLO exhibits higher absorbance than SLH, suggesting a lower content of these functional groups in the lignin sample derived from hydrolyzed sawdust, which is consistent with the results shown in Table 1. The bands at 1596 cm^−1^, 1512 cm^−1^, and 1420 cm^−1^ are assigned to skeletal vibrations and C=O group stretching in the syringyl and guaiacyl aromatic rings. The relatively higher intensity of the band at 1512 cm^−1^ compared with that of the band at 1595 in both spectra is attributed to the features of hardwood lignin structure. The bands at 1323 and 1215 cm^−1^ correspond to the syringyl structures of lignin. The most significant difference in the FTIR spectra of the lignin samples is observed in the range of absorbance bands of 1112–800 cm^−1^. These bands are attributed to C-C ring vibrations, overlapped with the absorbance of C-O vibrations in aliphatic hydroxyl and ether C-O-C groups. The intensity of the absorbance bands in this band range is remarkably higher for SLO than in the case of SLH. A decrease in the intensity of the absorbance bands, which corresponds to the presence of aliphatic and phenolic hydroxyl groups, and ether bonds in SLH in comparison with SLO may also indicate its more cross-linked chemical structure.

**Figure 8:**
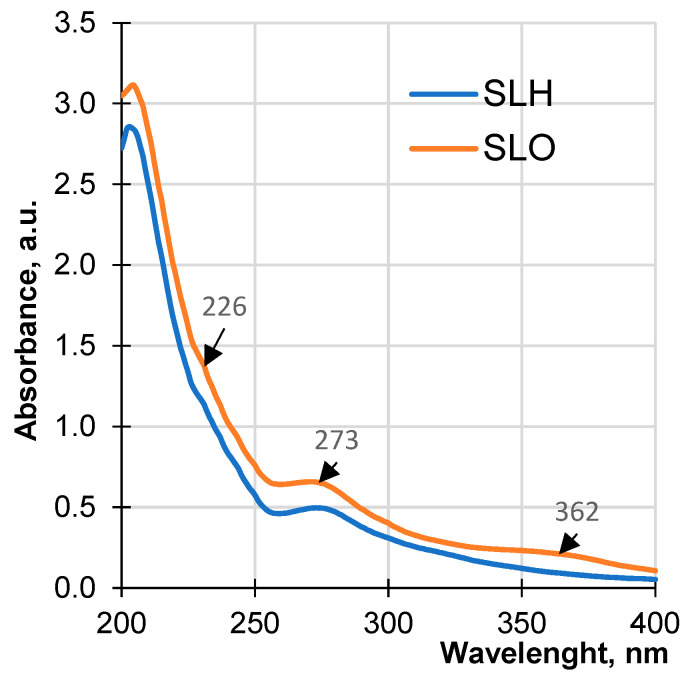
UV spectra of soda lignin samples obtained from original and pretreated sawdust.

Figure 8 shows the UV spectra of both soda lignin samples in an alkaline medium. The UV absorbance of the samples is conditioned by the ionization of non-etherified phenolic hydroxyl groups of phenylpropane structures containing α-carbonyl groups, hydroxystilbenes, and quinones [72]. The absorbance of SLO in the studied wavelength range is higher than the absorbance of SLH, which may be due to a more cross-linked polymer structure of the SLH molecules characterized by a lower phenolic hydroxyl group content.

A pronounced peak at 273 nm and a less pronounced shoulder at 227 nm are present in both UV spectra of the lignin samples. At the same time, a maximum at 362 nm assigned to carboxyl groups is absent in the UV spectra of SLH. Thus, the analysis of the results presented in Table 1 and the interpretation of the UV spectroscopic data can confirm a more condensed structure of SLH compared with that of SLO. The alkaline pretreatment loosens the structure of the lignocellulosic matrix by destroying the H-bonds network, partially removes hemicellulose due to disrupting ester bonds between lignin and hemicellulose, as well as cleaving ether groups in lignin in situ. It is assumed that the more open lignocellulosic structure of the hydrolyzed sawdust allows for a faster release of lignin fragments to the cooking solution, which promotes their higher concentration in comparison with the hydrolysis rate and the concentration of the lignin fragments released from the original sawdust. The enhanced concentration of the lignin fragments with the reactive groups, formed due to the partial breakdown of the lignocellulosic matrix, facilitated polycondensation reactions. One of the studied methods of polycondensation is via the addition reaction of lignin nucleophiles formed from the lignin fragments containing conjugated carbonyl structures—quinone methides, with the subsequent chemical conversions [73].

### 3.3. Aggregation of Lignin Samples

**Figure 9:**
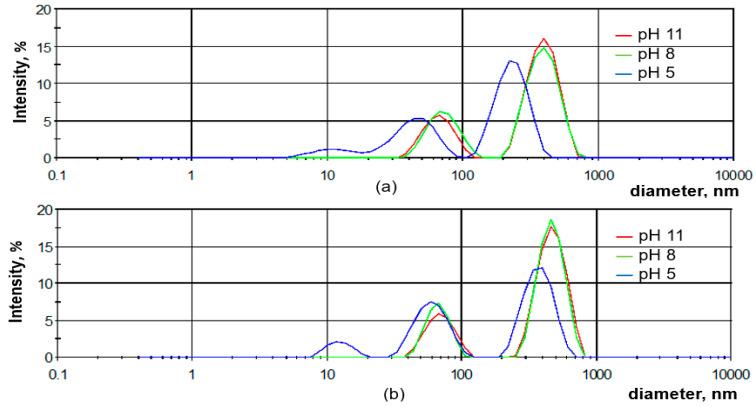
Size distribution of SLO (**a**) and SLH (**b**) particles depending on the pH aqueous solution.

**Figure 10:**
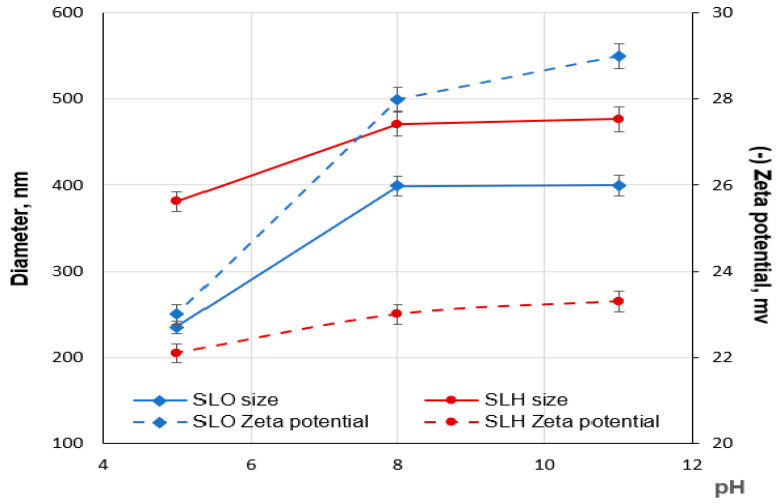
Dependence of size and zeta potential of SLO and SLH colloidal particles on pH aqueous solution.

The aggregation of the SLO and SLH particles in diluted aqueous solutions was studied, depending on pH, using a dynamic light scattering method. As shown in Figure 9, the aqueous solutions of both lignin samples are structured systems containing nano- (<100 nm) and colloidal (>100 nm) particles. Regardless of the pH, the average sizes of the SLH colloidal particles are larger than those of SLO, which may be attributed to the more cross-linked structure of the SLH molecules (Figure 10). The average negative ζ potential values are lower for the SLH particles than for the SLO particles, possibly due to a smaller content of acidic groups in the SLH molecules. With decreasing pH from 11 to 5, the average sizes of the colloidal particles drop from 477.3 nm to 381.1 nm and from 399.3 to 235.2 nm for SLH and SLO, respectively. The average negative ζ potential values for SLO particles decrease from 29.2 to −23.8 mV, but for SLH particles, the negative ζ potential values reduce from −23.3 mV to −22.7 mV. A polydispersity index (PDI) decreased from 0.38 to 0.24 and from 0.33 to 0.27 for the SLO and SLH particles, respectively, with dropping pH. A decrease in the average sizes as the pH drops is a result of the compaction of the lignin colloidal particles due to reducing electrostatic repulsion and strengthening π-π stacking interactions, increasing hydrogen linkages, and hydrophobic interactions.

According to Figure 10, the decrease in the average sizes and negative charge with dropping pH is more pronounced for the SLO colloidal particles than for the SLH ones, which can be explained by the different content of acidic groups (phenolic hydroxyl and carboxyl) in the lignin samples. At pH 5, the acidic groups in both soda lignin samples are mainly protonated, and the average zeta potential for both lignin colloidal particles becomes lower and closer in value. The significant effect of the acidic groups on the ζ potential values of the lignin particles confirms the validity of the proposed model of a lignin aggregate arrangement, according to which transformations in the lignin aggregates proceed at the “shell” level.

In strongly and moderately alkaline environments, the size distribution of the lignin particles for both samples displays a bimodal profile, indicating the presence of nano- and colloidal particles (Figure 9). In contrast, in an acidic medium, the size distribution of the SLO and SLH particles shows a polymodal character, with a third peak corresponding to nanoparticles with an average size of 11.5 to 12.5 nm.

**Figure 11:**
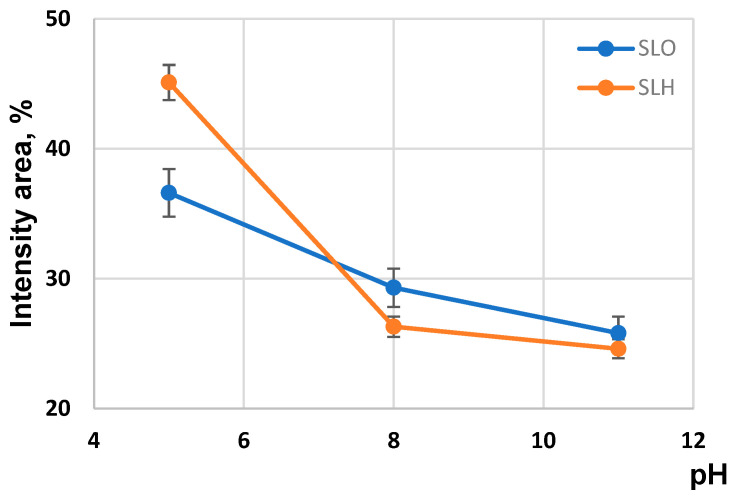
Size particle distribution for soda lignin samples obtained from origin and alkali-treated sawdust.

According to Figure 11, the intensity area associated with the presence of the nanoparticles during the transition from pH 11 to pH 5 enhances from 25.8% to 38.6% and from 24.6% to 45.1% for the SLO and SLH, respectively. At the same time, the intensity area for both lignin colloidal particles reduces. This may indicate the rearrangement in the lignin aggregates during the transition from an alkaline to an acidic medium. The phenomenon can be explained from the viewpoint of the existence of structural complementarity at the formation of the lignin self-assemblies. According to this suggestion, it can be assumed that the lignin nanoparticles with a defined construction and a defined set of functional groups can only participate in building the self-assemblies, which are the lignin colloidal particles. Sarkanen [40] previously suggested that lignin self-aggregates are complementary to each other in forming ordered supermolecular structures. Evidently, in our case, the excluded nanoparticles from the compacted colloidal structures do not respond to the defined criteria for inclusion in the lignin self-assemblies. The larger intensity area for the SLH excluded nanoparticles compared with the area for the SLO nanoparticles may be conditioned by the more branched structure of the SLH molecules due to the preferential polycondensation reactions at their primary formation.

### 3.4. Surface Activity

**Figure 12:**
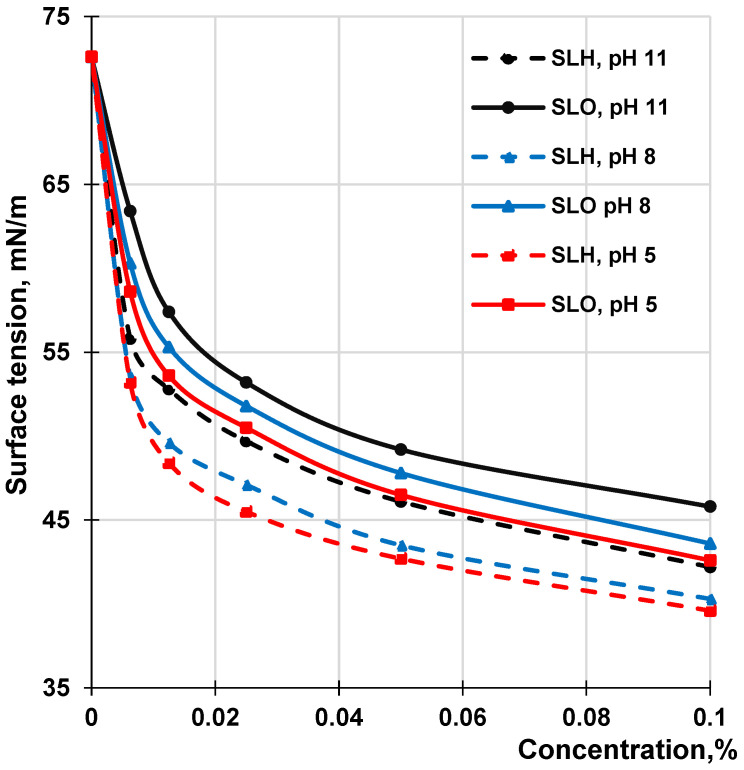
Surface tension of SLO and SLH solutions at the air–water interface depending on concentration at different pH.

It is known that the surface activity of a lignin molecule at various interfaces is determined by its chemical composition, affecting the hydrophilic–hydrophobic balance of the molecule, molecular weight, and conformation state, which affects the ability of the molecules to form monolayers at the interface. Figure 12 shows the adsorption isotherms at the air–water interface for the SLO and SLH aqueous solutions at different pH values in the range of concentrations that do not exceed 0.1%. Both lignin samples are characterized by relatively low surface tension values at the air–water interface. With decreasing pH, the surface tension for both lignin solutions reduces. A decrease in the surface tension with reducing pH can be explained by enhancing the hydrophobicity of the lignin molecules due to the protonation of phenolic hydroxyl and carboxylic groups. According to the adsorption isotherms, the significant decrease in surface tension occurs at the lowest concentrations, which correspond to the critical aggregation concentration (CAC) of lignin in the aqueous solutions. Further growth of the lignin concentration does not result in a remarkable drop in surface tension. At the CAC, the lignin samples at the air–water interface form the surface-ordered structures—the structured monolayers due to π-π stacking and hydrophobic and hydrogen bonding interactions. Due to the flexibility of β-O-4 alkyl ether linkages, the π-π stacking interactions between aromatic rings in different lignin molecules create surface layers at the air–water interfaces with closely matching thickness values [74]. It has been seen (Figure 12) that the SLO aqueous solution has higher surface tension values than the SLH solution at all concentrations and pHs. If the surface tension of water is a control point, a decrease in surface tension for a 0.1% solution ranges from 73% to 83% and from 59% to 72% for SLH and SLO, respectively, with a reduction in pH of the aqueous solutions from 11 to 5. The obtained CAC values for both lignin samples were between 0.08 g/L and 0.17 g/L; herewith, the CAC values for SLH were remarkably lower than in the case with the SLO, independent of pH. Since, in this range of concentrations, the lignin molecules of both samples are in aggregated form, it can be assumed that the surface layer can be formed by both the self-organized lignin molecules and their aggregates, as proposed in [55].

The adsorption capacity of molecules at the air–water interface can also be evaluated using a negative value of the tangent of the angle (−tg α). This angle is formed by a straight line that touches the isotherm curve at very low concentrations and intersects the concentration axis. The comparison of the tangent values of the angles (Figure 12) showed that, regardless of pH, their absolute magnitudes for the SLO adsorption isotherms were lower than those of the SLH adsorption curves. This confirms the enhanced surface activity of SLH compared to SLO, which was caused by shifting the hydrophilic–hydrophobic balance of its polymer structure toward hydrophobicity due to a lower content of the acidic groups in its composition. On the other hand, the higher content of the acidic groups in the SLO molecules in comparison with SLH can negatively affect the compatibility of the molecules at the formation of the surface layer due to electrostatic repulsion. This, in turn, can decrease the packing density of the surface layer and, therefore, increase the surface tension at the air–water interface.

## 4. Conclusions

The alkaline treatment of birch sawdust led to the remarkable weight loss due to the partial hemicellulose removal and reduced its mechanical strength. A decrease in the hydrolysate UV-adsorption with increasing alkali concentration was explained by the resorption of lignin fragments on the lignocellulose matrix. The yield of soda lignin and pulp in the case of the treated sawdust was 5.7% higher than in the case of the original sawdust. The alkaline treatment of the sawdust reduced the content of acidic groups and methoxyl groups in the extracted soda lignin compared with the chemical composition of the lignin from the original sawdust.

By analyzing the particle size distribution curves of the aqueous solutions of the soda lignin samples extracted from the original and hydrolyzed birch sawdust, it was found that, in alkaline medium, these solutions contain both lignin self-aggregates, represented by charged nanoparticles, and lignin self-assemblies, represented by charged colloidal particles. The modality of the lignin particle size distribution depends on the pH aqueous solution. In alkaline media, it displays a bimodal profile, indicating the presence of colloidal and nanoparticles. In an acidic medium, the size distribution for both lignin samples shows a polymodal character, with a third peak corresponding to the lignin molecule-sized moieties. Regardless of the pH, the average sizes of the colloidal particles of soda lignin from the hydrolyzed sawdust are larger than those for the particles from the original residue, which is attributed to a more cross-linked structure of the lignin molecules extracted from hydrolyzed sawdust.

The changes in the modality of the lignin particle size distribution, accompanied by the changes in the size and zeta potential with decreasing pH, indicated the rearrangement in the organization of the lignin particles during the transition from alkaline towards acidic medium. These observations can support the principle of structural complementarity, consisting of the selection of lignin structures with a specific conformation and a defined set of functional groups in building ordered supermolecular structures.

The surface activity of the lignin samples at the air–water interface was evaluated. The higher surface activity of the soda lignin extracted from the hydrolyzed sawdust, compared to the lignin from the original residue, was mainly attributed to a lower content of acidic groups in its chemical composition, shifting the hydrophilic–hydrophobic balance toward hydrophobicity.

The findings of this study can be utilized in developing new nano- and microscale value-added lignin products, which may be used for various applications, including drug delivery, the creation of functional bio-additives for polymer composite materials, and the production of lignin-based emulsifiers, stabilizers, and dispersants. Additionally, comprehending the self-organization behavior of lignin in aqueous medium can provide significant structural information that assists in optimizing pulping and extraction processes. This knowledge can also help select appropriate lignin modification reactions to enhance the efficiency of biorefining.

## Figures and Tables

**Table 1:** Functional compositions and yield of soda lignin samples.

Sawdust	Pulp		Soda Lignin						Yield, % from Wood	
	Yield, %	Lignin Content in Pulp, %	Yield, %	OCH_3_, %	Acid (OH) Groups, %			Klason Lignin, %	PULP + LIGNIN	Water-Soluble Destruction Products
					OH_all_	OH_fen_	OH_COOH_			
Origin	51.7	3.2	19.6	15.9	6.2	2.7	3.5	91.9	71.3	28.7
Hydrolysed	54.5	2.5	22.5	13.4	4.1	1.7	2.4	92.8	77.0	23.0

## Data Availability

Data are contained within the article.
